# HMMerge: an ensemble method for multiple sequence alignment

**DOI:** 10.1093/bioadv/vbad052

**Published:** 2023-04-17

**Authors:** Minhyuk Park, Tandy Warnow

**Affiliations:** Department of Computer Science, University of Illinois Urbana-Champaign, Urbana, IL 61801, USA; Department of Computer Science, University of Illinois Urbana-Champaign, Urbana, IL 61801, USA

## Abstract

**Motivation:**

Despite advances in method development for multiple sequence alignment over the last several decades, the alignment of datasets exhibiting substantial sequence length heterogeneity, especially when the input sequences include very short sequences (either as a result of sequencing technologies or of large deletions during evolution) remains an inadequately solved problem.

**Results:**

We present HMMerge, a method to compute an alignment of datasets exhibiting high sequence length heterogeneity, or to add short sequences into a given ‘backbone’ alignment. HMMerge builds on the technique from its predecessor alignment methods, UPP and WITCH, which build an ensemble of profile HMMs to represent the backbone alignment and add the remaining sequences into the backbone alignment using the ensemble. HMMerge differs from UPP and WITCH by building a new ‘merged’ HMM from the ensemble, and then using that merged HMM to align the query sequences. We show that HMMerge is competitive with WITCH, with an advantage over WITCH when adding very short sequences into backbone alignments.

**Availability and implementation:**

HMMerge is freely available at https://github.com/MinhyukPark/HMMerge.

**Supplementary information:**

Supplementary data are available at *Bioinformatics Advances* online.

## 1 Introduction

Multiple sequence alignment (MSA) is a necessary first step in many common bioinformatics pipelines, such as phylogenetic tree inference and metagenomic taxon identification, and so obtaining high quality MSAs has high relevance in downstream analyses (Matsen *et al.*, 2010; Morrison, 2006). One of the challenges in multiple sequence alignment is when the input sequence dataset has highly variable sequence lengths, a property that is found in many biological datasets (Nguyen *et al.*, 2015). Sequence length heterogeneity can be caused by evolutionary processes (e.g. large indels), but is also produced when datasets include reads generated by Illumina and other short read sequencing technologies (Shendure and Ji, 2008).

UPP (Nguyen *et al.*, 2015) and its successor WITCH (Shen *et al.*, 2022) are methods that are designed to align datasets when the input has sequence length heterogeneity. Both methods use a two-stage approach, where they first identify and extract a subset of the input sequences (based on their sequence length) that are considered to be ‘full-length’, and then align these sequences, forming the ‘backbone alignment’. Both then build an ensemble of profile Hidden Markov Models (eHMM) to represent the backbone alignment, where each profile HMM is built on a subset of the input sequences in the backbone alignment [see Durbin *et al.* (1998) for an introduction to profile Hidden Markov Models]. UPP adds each of the remaining sequences (called ‘queries’) to the backbone alignment, as follows: (i) each query sequence selects a best-fitting HMM from the eHMM based on the bit scores, (ii) the Viterbi algorithm is used to find an optimal path for the query sequence through the selected HMM and (iii) the match states in the optimal path define an alignment of the query sequence to the backbone alignment, which is used to add the query sequence into the backbone alignment. WITCH elaborates on this approach by weighting each profile HMM with the probability it has of generating the query sequence, and then combines the optimal alignments (one such alignment for each profile HMM in the eHMM) into a consensus alignment using the Graph Clustering Merger from Smirnov and Warnow (2021a). Both UPP and WITCH produce more accurate alignments than standard methods, including MAGUS (Smirnov and Warnow, 2021a), when datasets have fragmentary sequences (Nguyen *et al.*, 2015; Shen *et al.*, 2022), and WITCH generally dominates UPP for accuracy (Shen *et al.*, 2022).

Here, we present HMMerge, a new approach for computing alignments. We follow the same initial steps as WITCH and UPP (i.e. we use the same technique to build the backbone alignment and the ensemble of HMMs representing the backbone alignment). We then deviate from WITCH in several ways. First, we use only a selected subset of the HMMs from the WITCH ensemble of HMMs for the HMMerge ensemble. Second, instead of aligning the query sequence to each HMM and then computing a consensus of these extended alignments, we use the HMMerge ensemble of HMMs to build a new HMM (not a standard profile HMM, however) to represent the backbone alignment. The topology of the HMM is the same for all query sequences but the numeric parameters (transition and emission probabilities) are derived for each query sequence. Finally, given a query sequence, the numeric parameters are computed and then used to align the query sequence to the backbone alignment.

As this study shows, using both simulated and biological data, HMMerge is competitive with WITCH when aligning datasets with sequence length heterogeneity (sometimes more accurate and sometimes less accurate), and has an advantage in some cases when there are very short sequences in the dataset. More generally, we show that two-stage methods, including the use of MAFFT-addfragments (Katoh and Frith, 2012), provide more reliable accuracy when aligning datasets with sequence length heterogeneity than standard methods.

## 2 Methods

We first describe HMMerge, which is our new approach for aligning sequence datasets that exhibit sequence length heterogeneity. We then describe the experimental study we used to evaluate HMMerge.

### 2.1 HMMerge

HMMerge operates in three stages: the backbone stage, the construction of the eHMM for the backbone, and then the search-and-align stage. HMMerge uses the same process for stages 1 and 2 as handled by both UPP and WITCH, but then differs from both for the third stage. Due to space limitations, we briefly review the first two stages, and provide details only for the third stage for HMMerge; see Shen *et al.* (2022) for additional details about how these first two stages are performed in WITCH.

#### 2.1.1 Stage 1: computing the backbone alignment

In the backbone stage, the input sequences are split into two sets, with one set containing some of the ‘full-length’ sequences and the remaining sequences denoted ‘query’ sequences. An alignment is built on the full-length sequences, using external methods [e.g. PASTA (Mirarab *et al.*, 2015) or MAGUS]. However, any method can be used to produce the backbone alignment, and the use of the Bayesian method BAli-Phy (Suchard and Redelings, 2006) for statistical alignment was studied in this context (Nute and Warnow, 2016).

#### 2.1.2 Stage 2: computing the eHMM

Once the backbone alignment is computed, a tree is computed on the backbone alignment [e.g. using a maximum likelihood method, such as FastTree 2 (Price *et al.*, 2010) or RAxML (Stamatakis, 2014)]. Then, the backbone tree is decomposed into subsets by edge deletions (removing ‘centroid edges’ that split the leaf-set into two roughly equally sized parts) until each subset is at most a given size specified by the user. For HMMerge, we stopped at subset size 50, for the sake of runtime (see Supplementary Materials Section S5). The full set of backbone sequences and any subset produced by the decomposition pipeline becomes one of the subsets. A profile HMM is constructed on each subset using *hmmbuild* from the HMMER suite (Eddy, 2011). The collection of profile HMMs produced in this stage is referred to as an ‘ensemble of HMMs’ for the backbone alignment, or ‘eHMM’.

#### 2.1.3 Stage 3: search-and-align stage

For this stage, HMMalign builds a new HMM (not a profile HMM, however), based on a selected subset of the HMMs in the ensemble. Here, we explored several options for the selection. The default case is where we use just the HMMs based on the minimal sequence subsets, which are pairwise-disjoint; we refer to this as the Disjoint(50) eHMM. We also explore Disjoint(50)+BB, which is where we add the HMM for the full backbone alignment to Disjoint(50). Finally, we explore UPP(50), which is where we use the same ensemble as used by UPP decomposing to size 50, which is based on a hierarchical set of sequence subsets. Henceforth, when we refer to ‘eHMM’, we mean whichever eHMM has been selected, but the default in our experiments is to use Disjoint(50).

To add a given query sequence into the backbone alignment using HMMalign, each query sequence scores each HMM in the eHMM according to an ‘adjusted’ bit score, which is designed to be an estimate of the probability that the HMM generated the given query sequence [see Shen *et al.* (2022) for the derivation of the adjusted bit score]. Note that the choice of eHMM affects the adjusted bitscore calculation, since these depend on whether or not the subsets are disjoint, as described in Shen *et al.* (2022).

### 2.2 Merged HMM construction

HMMerge computes the topology of the merged HMM from the ensemble of HMMs, and each query sequence then defines the numeric parameters on this common topology. Here we describe first how we compute the topology, and then how we define the numeric parameters.

Since every HMM in the ensemble is constructed for a sub-alignment of the backbone alignment (induced by a subset of the rows of the alignment matrix), each match state in each of these HMMs corresponds to a specific column (also known as ‘site’) in the backbone alignment. The merged HMM will have the same set of states (match state, insertion states and deletion states) as the HMM for the backbone alignment, but there may be additional edges in the merged HMM, as we now describe. Suppose for example one of the subset alignments is entirely gapped in sites 10 through 20, but otherwise all the sites have at least one letter and so are not entirely gapped. For this subset alignment, the HMM will have match states for all sites in the backbone alignment other than those for sites 10 through 20. Therefore, the HMM for this subset alignment will have a transition from the match state for backbone alignment site 9 to the match state for backbone alignment site 21, as well as other transition edges. These transition edges will be included in the merged HMM topology. As a result, the merged HMM is not a standard profile HMM.

Although the topology remains fixed for each query sequence, the numeric parameters (i.e. transition probabilities and emission probabilities) depend on the query sequence. Each query sequence assigns a weight to each HMM using the adjusted bitscore, which reflects the probability that the HMM produced the query sequence; see Shen *et al.* (2022). In the merged HMM for that given query sequence, we then use these bit scores to compute the numeric parameters, as follows. To compute the transition probability on an edge from state A to state B, we compute the weighted sum of the transition probabilities on the same edge across all HMMs that have that edge, where the weight for a given HMM is the adjusted bit score of that HMM for that query sequence. Similarly, the emission probabilities for a match state are computed by the weighted sum of the emission probabilities for that match state across the HMMs that have that match state.

Given the ensemble of HMMs and a single query sequence, we use this process to compute the merged HMM. Note that the HMMs for the different query sequences differ only in the numeric parameters, and that the topology of the merged HMMs has all the edges from the HMMs in the ensemble.

### 2.3 Modifying the merged HMM to enable glocal alignment

Once the merged HMM is defined for the given query sequence, we use a standard Viterbi algorithm to align the query sequences to the merged HMM. This alignment defines an extended alignment of the query sequence to the backbone alignment in the usual way. However, since the Viterbi algorithm is naturally a global alignment algorithm, we introduce additional edges to the merged HMM in order to enable a glocal alignment.

A glocal alignment is a hybrid approach in which the entirety of one sequence is aligned to a segment from another sequence. An example where this may be useful is aligning a short sequence to a long sequence. In this case, we would like to align the entirety of the short sequence to a specific region in the longer sequence. In order to allow for glocal alignment without incurring any penalties, i.e. aligning a query sequence to a specific region of the backbone alignment, we need to allow for our HMM algorithm to be able to start at any match state and end at any match state.

**Fig. 1.**
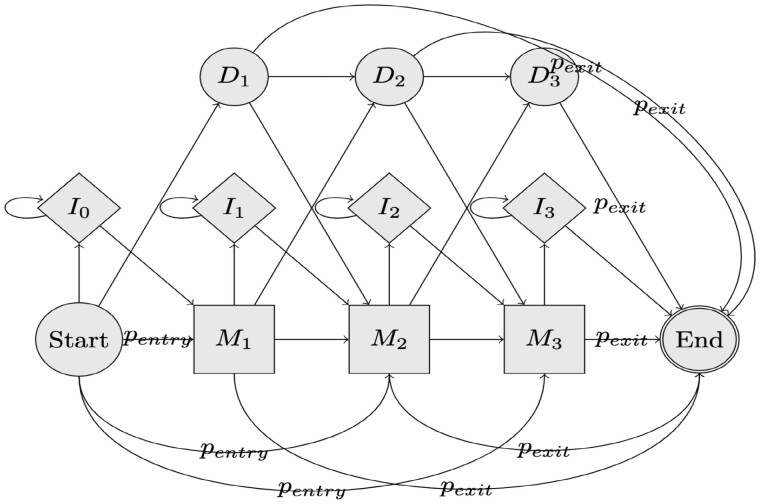
Equal entry exit HMM example. Here, we show a visualization of an example HMM with equal entry and exit probability edges. $p_{\text{entry}}$ and $p_{\text{exit}}$ represent the probabilities of a path taking that edge. $M_{i}$ stands for the *i*th match state, $I_{i}$ stands for the *i*th insertion state, and $D_{i}$ stands for the *i*th deletion state. Only the insertion and match states emit letters

We define two new probabilities, $p_{\mathrm{entry}}$ and $p_{\mathrm{exit}}$, which represent the transition probabilities from the start state to each match state and from each match and delete states to the end state, respectively; see Figure 1. Let *S* be the number of match states. $p_{\mathrm{exit}}$ is a constant 0.1 whereas $p_{\mathrm{entry}}$ is $\frac{0.1}{S}$. To account for the new edges, we normalize the edge weights such that all outgoing edge weights sum to 1 for each state.

### 2.4 Combining the extended alignments

After all query sequences are added to the backbone alignment, the final alignment is produced through transitivity (this is the same technique as used in UPP and WITCH). Note however that homologies in the final alignment only take place through the match states—letters that are introduced through insertion states are never considered homologous to any other letters. These letters that are added through insertion states are indicated through the use of lower-case letters in the output alignment.

## 3 Experimental study design

We performed a sequence of experiments. In the first two experiments, we explored HMMerge in comparison to other methods using the default eHMM, Disjoint(50). We then performed an additional experiment where we varied the selection of HMMs for the ensemble. While we used some standard methods [e.g. MAFFT (Katoh *et al.*, 2002) and MUSCLE (Edgar, 2004)], a major focus of this study is on two-stage methods, like HMMerge, that operate by constructing a backbone alignment and then adding the remaining sequences into the backbone alignment. Thus, we also used UPP, WITCH and the use of MAFFT-addfrags in this study. On all datasets tested, these four two-stage methods used the same backbone alignments computed by MAGUS and backbone trees computed by FastTree (Price *et al.*, 2010). All the tested datasets exhibit sequence length heterogeneity, as shown in Supplementary Figures S10–S14.

### 3.1 Experiment 1: analyses of simulated datasets with introduced fragmentation

In Experiment 1, we explored both standard and two-stage methods on simulated datasets with introduced fragmentation.

### 3.2 Experiment 2: analyses of biological datasets

In this experiment, we examined both standard and two-stage methods on five rRNA datasets from the Comparative Ribosomal Website (CRW) (Cannone *et al.*, 2002).

### 3.3 Experiment 3: exploring results with changes to the eHMM

In the previous two experiments, we used Disjoint(50) for the eHMMs we used for HMMerge. In this experiment, we explored the impact of using a larger eHMM –that is, Disjoint(50)+BB (i.e. including the HMM for the backbone alignment) or UPP(50) [i.e. using all the HMMs in the UPP(50) ensemble]. We perform this experiment on a subset of the study datasets.

### 3.4 Simulated datasets

We created high-fragmentary (HF) versions of simulated nucleotide datasets, each with 1000 sequences, using three different simulators: ROSE (Stoye *et al.*, 1998), RNASim (Mirarab *et al.*, 2015) and INDELible (Fletcher and Yang, 2009). To make these HF versions, we used the same technique and parameters as in Smirnov and Warnow (2021b). These HF datasets have 1000 sequences, and half of the sequences have been trimmed (i.e. a prefix and suffix are deleted) to produce a substring that is on average 25% of the median length with a standard deviation of 60 base pairs. These datasets consist of 500 full-length sequences and 500 fragmentary sequences.

The ROSE simulated datasets have 15 model conditions each with 20 replicates, where each model condition varies by the average length of gaps (‘S’ for Short, ‘M’ for Medium or ‘L’ for Long) and by their varying substitution rates (models are numbered 1 through 5, and the substitution rate decreases as the number increases).

The RNASim simulated datasets were originally used to evaluate alignment methods in Mirarab *et al.* (2015), and the simulation protocol for generating these datasets is described in Appendix A in the supplementary materials for Mirarab *et al.* (2015). These sequences evolve under a biophysical model that enforces selection in order to maintain RNA secondary structure. We use HF versions from Smirnov and Warnow (2021b) and we also made an ultra-high fragmentary, or ‘UHF’, version of the dataset by setting the target mean to be exactly half of the HF condition. The target mean sequence length was 388 for the HF condition and 194 for the UHF condition. The standard deviation remained at 60, which is the same as the original study. We used 10 replicates each for both datasets.

The INDELible datasets were created for this study. The model tree for the simulation was taken from the set of gene trees from the ASTRAL-II study (Mirarab and Warnow, 2015). In order to make alignment estimation challenging, we scaled the branch lengths of the model tree by a factor of two. Since these simulated datasets evolve without indels, we modified the simulation to produce indels. The indel gap length distribution was taken from the PASTA study (Mirarab *et al.*, 2015), where INDELible was used to generate simulated datasets. We simulated sequences under these conditions with an indel rate of 0.001 and 0.005, relative to a substitution rate of 1. These sequences were then fragmented according to the HF protocol described above. 10 replicates were created for each indel rate model condition.

### 3.5 Biological datasets

**Fig. 2.**
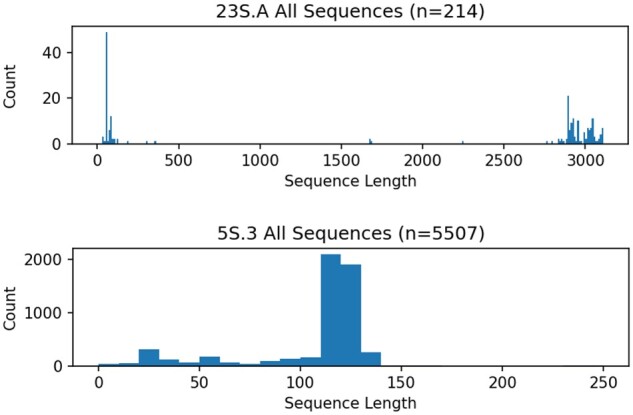
Sequence length heterogeneity histograms for two biological datasets (23S.A and 5S.3) from the Comparative Ribosomal Website (Cannone *et al.*, 2002)

We used datasets from the Comparative RNA Website (CRW) (Cannone *et al.*, 2002), which have curated alignments based on rRNA structure for the 23S and 5S ribosomal RNA genes. We chose 23S.A, 23S.C 5S.3, 5S.E and 5S.T for our study. These datasets vary in how much sequence length variability they have, with 23S datasets exhibiting more fragmentation than the 5S datasets (Fig. 2).

To separate the biological datasets into backbone sequences and query sequences, we split the sequences using visual inspection of their sequence length histograms. As a result, 23S.A and 23S.C were both split at 1250 (so that sequences shorter than 1250 in length were considered query sequences and the rest were backbone sequences), and 5S.3, 5S.E, and 5S.T were split at 100 (with the corresponding definition).

For the simulated datasets, we summarize the empirical properties in Supplementary Table S1. For the CRW biological datasets, we summarize the empirical properties in Table 1 and Supplementary Table S2.

**Table 1.** Biological DNA/RNA dataset overview

Name	No. of sequences	Avg. *p*-dist.	Avg. len.
23S.A	214	0.293	1851
23S.C	374	0.143	2087
5S.3	5507	0.417	106
5S.E	2774	0.305	96
5S.T	5751	0.425	106

*Note*: Here, we show the basic empirical statistics about the datasets used in this study. *p*-dist. refers to the normalized Hamming distance [i.e. the number of positions where the two sequences have different letters divided by the number of positions where both sequences have letters (are not gapped)].

### 3.6 MSA methods

We compare our new MSA pipeline, HMMerge, to three other two-stage methods (UPP, WITCH and MAFFT-addfragments), each using the same backbone alignment. We also compare these two-stage methods to a collection of leading MSA methods, including MAGUS, PASTA, Clustal Omega (Sievers *et al.*, 2011), MUSCLE (Edgar, 2004), MAFFT (Katoh *et al.*, 2002) and T-COFFEE (Garriga *et al.*, 2019). The only methods that were not run in their default modes were MAFFT, which was run using the L-INS-i algorithm (to maximize accuracy), and T-COFFEE, which was run using the regressive mode. The exact commands and versions for each of the methods are supplied in the Supplementary Materials Section S1.

### 3.7 Computational resources

All runs of HMMerge, WITCH, UPP and MAFFT-addfrags were run on the Illinois Campus Cluster with 16 cores available for parallelism and a default memory limit of 64GB. The standard methods (MAGUS, PASTA, MAFFT, Clustal Omega, T-COFFEE and MUSCLE) were run on BlueWaters (Bode *et al.*, 2013) for the ROSE and CRW datasets. However, as discussed in Supplementary Materials Section S5, HMMerge needed extra memory for some analyses, which required us to use a high-memory machine with up to 1TB of memory for these analyses.

### 3.8 Evaluation criteria

Alignment error was calculated using FastSP (Mirarab and Warnow, 2011). SPFN and SPFP are based on pairwise homologies, i.e. pairs of letters that appear in the same column of the estimated or reference alignment. Thus, SPFN, or sum-of-pairs false negatives, is the fraction of true pairwise homologies (i.e. homologies in the reference alignment) that are not found in the estimated alignment, while SPFP, or sum-of-pairs false positives, is the fraction of putative homologies in the estimated alignment that are not in the reference alignment. Note that letters that are emitted by our HMM-based alignment method through insertion states are never considered homologous to any other letters, and are represented by lower-case letters in the output alignment.

We include both standard methods (i.e. methods that do not operate in two stages) as well as two-stage methods. When we include standard methods in the comparison, we report ‘total’ alignment error, which is alignment error on the entire sequence dataset. However, when we compare two-stage methods to each other, we report ‘query-only’ alignment error. This allows us to take advantage of the fact that all the two-stage methods are using the same backbone alignment.

## 4 Results

### 4.1 Experiment 1: analyses of simulated datasets with introduced fragmentation

#### 4.1.1 ROSE-HF nucleotide simulated datasets

The comparison of six standard methods (MAGUS, PASTA, T-COFFEE, MUSCLE, MAFFT-linsi and Clustal-Omega) and UPP on the 15 ROSE model conditions is given in Supplementary Figure S1. While all methods have nearly the same accuracy on the easier model conditions, the gap between methods increases with the difficulty of the model condition. UPP was consistently the method with the least error on the harder model conditions, and for the three hardest model conditions (1000L3-HF, 1000S1-HF and 1000M1-HF) there is a gap of more than 0.1 in average alignment error between UPP and the next best method, which is MAGUS.

Supplementary Figure S2 compares HMMerge, WITCH and UPP for query-only alignment error across the nine hardest model conditions. In this comparison, UPP always has the highest average query-only error, and HMMerge is always at least as accurate as WITCH. However, the gap between WITCH and HMMerge is never more than 0.020. Also, MAGUS has higher error than these three two-phase methods (HMMerge, WITCH and UPP), as seen in Supplementary Figure S3.

**Fig. 3.**
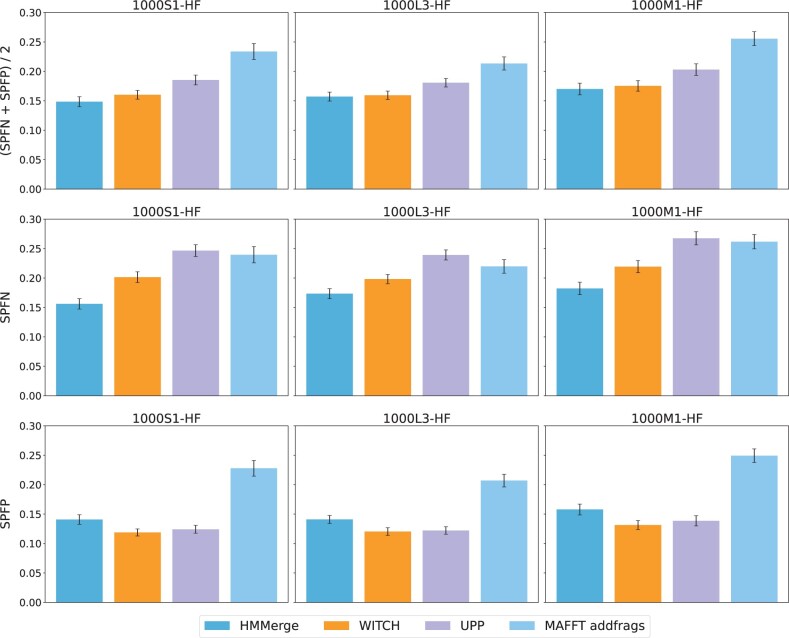
Query sequence alignment error of two-stage methods on ROSE simulated datasets with introduced fragmentation. Each model condition has 20 replicates; error bars show standard error

A comparison between all four two-stage methods on the three hardest model conditions, 1000S1-HF, 1000L3-HF and 1000M1-HF (Fig. 3), shows that HMMerge and WITCH have the lowest average error (and never differ by more than 0.012), followed by UPP, and finally by MAFFT-addfrags.

#### 4.1.2 RNASim1000-HF and RNASim1000-UHF datasets

A comparison between the standard methods and the two-stage methods is shown in Supplementary Figure S6. The four two-stage methods, MAGUS and MAFFT-linsi have average alignment error between 0.101 and 0.127 while the remaining methods have average alignment error that is never less than 0.250. MAGUS and MAFFT-linsi always have higher average alignment error than the four two-stage methods, and the difference is always at least 0.007.

Comparing the four two-stage methods to each other with respect to query-only alignment error (Table 2 and Supplementary Fig. S5), we see the following trends. UPP, WITCH and HMMerge never differ by more than 0.005 for average alignment error and MAFFT-addfrags always has higher average alignment error. For example, on the HF condition, MAFFT-addfrags has 0.123 average alignment error and the other three methods have average error between 0.100 and 0.101. On the UHF condition, MAFFT-addfrags has 0.126 average alignment error and the other three methods have average error between 0.103 and 0.108. In sum, UPP, WITCH and HMMerge are very close in accuracy, and all are somewhat more accurate than MAFFT-addfrags.

**Table 2.** Query sequence alignment error of two-stage methods on RNASim1000 with introduced fragmentation

RNASim1000	HMMerge	WITCH	UPP	MAFFT addfrags
HF—average	0.100	0.101	0.100	0.123
HF—SPFN	0.102	0.103	0.108	0.126
HF—SPFP	0.098	0.099	0.092	0.120
UHF—average	0.103	0.105	0.108	0.126
UHF—SPFN	0.107	0.108	0.123	0.128
UHF—SPFP	0.099	0.102	0.093	0.124

#### 4.1.3 INDELible datasets

Results on the INDELIBLE datasets, shown in Supplementary Figure S4, show that the lowest average alignment error achieved by a standard method on the 0.001-HF condition was 0.102, achieved by MAGUS, with PASTA at 0.105; MAFFT-linsi was higher at 0.212, and all the other standard methods had errors at least 0.309. In contrast, all four two-stage methods had error rates between 0.072 and 0.083. The trends on the 0.005-HF condition were similar, with the lowest average alignment error of a standard method achieved by MAGUS at 0.353 (with the next lowest error at 0.479, achieved by MAFFT-linsi). In contrast, the four two-stage methods had average alignment error between 0.279 and 0.295. Thus, for the INDELIBLE model conditions, there was a clear gap between the two-stage methods and the standard methods.

**Fig. 4.**
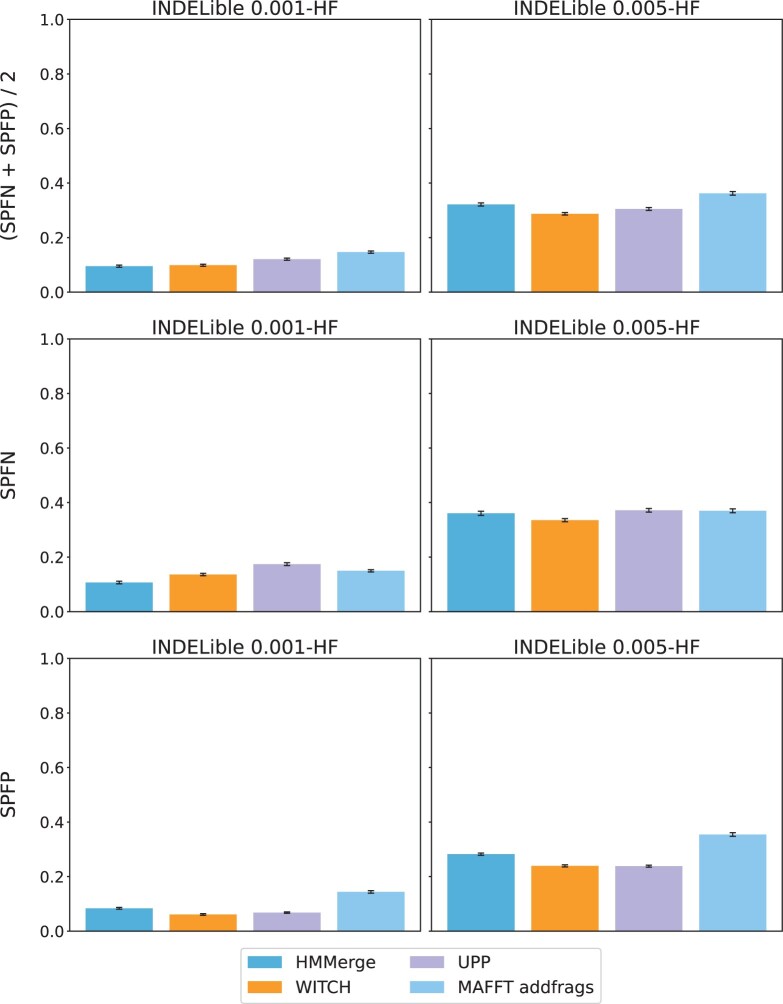
Query sequence alignment error of two-stage methods on INDELible simulated datasets with introduced fragmentation. The model conditions have 10 replicates; error bars show standard error

We compare the four two-stage methods with respect to query-only alignment error in Figure 4. MAFFT-addfrags has higher average alignment error than the other three methods; for example, it has error of 0.147 on the 0.001-HF condition compared to the second highest error of 0.121, attained by UPP, and error of 0.326 on the 0.005-HF condition compared to the second highest error of 0.321, attained by HMMerge. Overall, MAFFT-addfrags has higher average error on these conditions than the other three two-stage methods, but differences between the four two-stage methods are generally small.

### 4.2 Experiment 2: results on biological datasets

A comparison of all methods, including the standard methods, is given in Supplementary Figure S8. Error rates are highest on 5S.3 and 5S.T, compared to the 23S datasets and 5S.E. However, based on this figure, it is clear that MUSCLE, T-COFFEE and Clustal-Omega have substantially higher average error than the other alignment methods. Average error for the remaining methods (the four two-stage methods, MAGUS, MAFFT-linsi and PASTA) are generally close, but with an advantage to the two-stage methods, as we now describe.

On the 23S.A dataset, MAFFT-linsi and MAGUS have the lowest error (0.074), followed closely by the four two-stage methods at 0.076–0.077. On the 23S.C dataset, MAFFT-linsi has the lowest error (0.038), followed closely by the four two-stage methods at 0.039, and by MAGUS at 0.040. On the 5S.E dataset, MAFFT-linsi has the lowest error (0.020), followed closely by the four two-stage methods and MAGUS at 0.024–0.029. On the 5S.3 dataset, MAFFT-addfrags has the lowest error (0.096), followed closely by the other three two-stage methods (0.099–0.101); the lowest error for any standard method is 0.105, achieved by PASTA. Finally, on the 5S.T dataset, MAFFT-addfrags has the lowest error (0.097), followed closely by the other three two-stage methods (0.099–0.102); the lowest error for any standard method is 0.108, achieved by MAGUS. We also explored the impact of using MAFFT-linsi for the backbone alignment instead of MAGUS on these datasets; as seen in Supplementary Figure S9, this reduced accuracy rather than improving it. Finally, it is worth noting that MAFFT-linsi was the most accurate on three datasets (23S.A, 23S.C and 5S.E), and although it did not fall in the top category for the two remaining datasets, 5S.T and 5S.3, it fell in the middle of the methods with respect to accuracy.

With the exception of MAFFT-linsi (and perhaps MAGUS, which also generally came close to the four two-stage methods), the trends on these five CRW datasets show that the four two-stage methods reliably have lower average alignment error than the standard methods.

A comparison of the four two-stage methods with respect to query-only alignment error (Table 3 and Supplementary Fig. S5) show that no single two-stage method reliably was more accurate than the others, but we note that HMMerge specifically was less competitive on the 5S datasets than it was on the 23S datasets.

**Table 3.** Query sequence alignment error of two-stage methods on CRW biological datasets

	HMMerge	WITCH	UPP	MAFFT addfrags
23S.A—average	0.084	0.121	0.107	0.091
23S.A—SPFN	0.103	0.174	0.148	0.105
23S.A—SPFP	0.066	0.068	0.065	0.077
23S.C—average	0.039	0.037	0.035	0.040
23S.C—SPFN	0.039	0.036	0.036	0.039
23S.C—SPFP	0.039	0.038	0.035	0.041
5S.3—average	0.106	0.087	0.096	0.050
5S.3—SPFN	0.129	0.120	0.138	0.059
5S.3—SPFP	0.084	0.053	0.055	0.042
5S.E—average	0.091	0.060	0.083	0.068
5S.E—SPFN	0.103	0.077	0.120	0.071
5S.E—SPFP	0.079	0.042	0.045	0.064
5S.T—average	0.106	0.076	0.096	0.053
5S.T—SPFN	0.122	0.093	0.129	0.054
5S.T—SPFP	0.090	0.058	0.062	0.051

### 4.3 Experiment 3: impact of changing the eHMM

Inspired by results reported in Nguyen *et al.* (2015) that showed that using hierarchical ensembles instead of disjoint ensembles improved accuracy, we explored changes to the eHMM we use in HMMerge to see if adding HMMs would improve accuracy. Recall that by default, HMMerge uses Disjoint(50). In this experiment, we also explore the use of Disjoint(50)+BB [i.e. adding the HMM for the full backbone alignment to Disjoint(50)] as well as using the entire UPP(50) ensemble. We explored this partially for some simulated datasets as well.

On the three CRW 5S datasets (Supplementary Table S3), Disjoint(50) and Disjoint(50)+BB had the same alignment error. The same was true for two simulated datasets (INDELible 0.001-HF and ROSE 1000S1-HF). On the other hand, using the UPP(50) eHMM improved accuracy compared to Disjoint(50). Specifically, average alignment error dropped from 0.106 to 0.102 for the 5S.3 dataset, from 0.91 to 0.89 for the 5S.E dataset, and from 0.106 to 0.096 for the 5S.T dataset.

Thus, this experiment suggests the possibility that HMMerge accuracy could improve by using larger ensembles [i.e. adding HMMs to the Disjoint(50) ensemble], but also suggests that it is not sufficient to simply add the HMM for the backbone alignment. See Supplementary Materials Section S4 for additional results and discussion.

## 5 Discussion

This study introduced HMMerge, a new technique for aligning datasets that exhibit sequence length heterogeneity. Like its predecessors, WITCH and UPP, it operates by constructing a backbone alignment of full-length sequences and then adds the remaining sequences into the backbone alignment using a method based on hidden Markov models. This study showed that HMMerge, WITCH and UPP all tended to have close accuracy, and generally that HMMerge and WITCH were more accurate than UPP. We also observed that using MAFFT-addfrags to add the remaining sequences into the backbone alignment had comparable accuracy to HMMerge, WITCh and UPP under some conditions. Finally, we saw that standard methods (i.e. methods that do not operate in this two-stage manner) tend to have lower accuracy than two-stage methods.

It is interesting however to consider the difference between trends observed on the five biological datasets and the simulated datasets. Specifically, for the simulated datasets, we saw generally that the four two-stage methods had reliably lower error than the standard methods, except when the mutation rate was low (as indicated by a low average *p*-distance). The biological CRW datasets have generally lower *p*-distances than the harder simulated model conditions, and on these biological datasets, we saw that MAFFT-linsi was the most accurate method on three of the datasets and that MAGUS also was close to the two-stage methods in accuracy. Thus, the likely explanation for this difference may be the mutation rate, as the conditions where the two-stage methods had clear dominance over the standard methods had high rates of evolution as well as substantial fragmentation. Thus, it may be that two-stage methods may only be needed when datasets exhibit both these factors: substantial sequence length heterogeneity and also large *p*-distances.

It is also interesting to consider why sometimes, but not always, HMMerge is more accurate than WITCH. Based on the trends shown in the simulated datasets and the contrast between its performance on the 23S and 5S biological datasets, we conjecture that HMMerge may provide an advantage under conditions where there are many very short sequences. If this is the case, the choice between WITCH and HMMerge might be based on the type of sequence length heterogeneity in the dataset.

This study suggests several directions for future research. Clearly, HMMerge is more computationally intensive to use than WITCH or UPP (Supplementary Materials Section S5); hence, improving its implementation is necessary. However, changes to the design may also yield improved accuracy, as the preliminary results we obtained in modifying HMMerge through changes to its eHMM [and especially through the use of the UPP(50) eHMM] suggest.

From an algorithms design perspective, WITCH and HMMerge both benefit from the use of adjusted bit-scores, which is an innovation relative to UPP. They also both use the same ensemble of HMMs for the representation of the backbone alignment, but beyond this they differ. WITCH computes an extended alignment from each of the profile HMMs for a given query, and then uses a graph-based algorithm to combine these extended alignments into a single extended alignment. In contrast, given the query sequence, HMMerge produces a new HMM with a topology that has the potential to differ from that of the canonical profile HMM topology used for each HMM in the given eHMM, and then uses that new HMM to align the query sequence. This new profile HMM creation is the novel algorithmic aspect of HMMerge. As we demonstrated in this study, this machine learning model provides an advantage over WITCH in some cases. Future research should explore additional algorithm design strategies to determine how to best take advantage of this insight.

## 6 Conclusion

HMMerge is a multiple sequence alignment method that was designed to address the challenge of aligning datasets that exhibit high levels of sequence length heterogeneity. HMMerge operates in two stages, where the first stage extracts and aligns a subset of the sequences it considers to be full-length (thus producing a backbone alignment), and in the second stage it adds the remaining sequences into the backbone alignment. HMMerge builds on techniques in previous methods that use ensembles of profile HMMs to represent the backbone alignment, but does so by creating a new HMM with more edges than a standard profile HMM. As our study shows, this novel machine learning model enables HMMerge to improve on WITCH when adding very short sequences into the backbone alignment, and is otherwise competitive with WITCH (sometimes better, sometimes not as good).

There are implications of this study for both biologists and algorithms developers. For the biologists, the implications are that when aligning datasets with fragmentary sequences, standard methods (such as MAFFT, T-COFFEE, MUSCLE, Clustal Omega, etc.) may not provide good accuracy, and instead alignment methods that are designed for use on such datasets should be considered. Here we would recommend the use of a two-stage method, though the conditions under which each such method might provide an advantage over the others are not yet understood.

For the algorithms designer, this study shows the potential benefits to be gained from novel uses of ensembles of profile Hidden Markov Models to represent backbone alignments. Furthermore, although WITCH and HMMerge both provide improved accuracy compared to UPP, which was the first method in this category, it seems very likely that additional algorithmic exploration could lead to improved accuracy and potentially faster methods. We hope that this study will lead to new algorithmic advances, in order to best improve alignment accuracy under conditions with sequence length heterogeneity, since these are increasingly common in biological datasets.

## Supplementary Material

vbad052_Supplementary_Data

## Data Availability

HMMerge is freely available at https://github.com/MinhyukPark/HMMerge. The INDELible datasets are available at the Illinois Data Bank (Park and Warnow, 2023).

## References

[vbad052-B1] Bode B. et al (2013) The Blue Waters Super-System for Super-Science. In: Vitter, J. (ed.) Contemporary High Performance Computing. Chapman and Hall/CRC, New York, pp. 339–366.

[vbad052-B2] Cannone J.J. et al (2002) The comparative RNA web (CRW) site: an online database of comparative sequence and structure information for ribosomal, intron, and other RNAs. BMC Bioinformatics, 3, 1–31. 11869452 10.1186/1471-2105-3-2 PMC65690

[vbad052-B3] Durbin R. et al (1998) Biological Sequence Analysis: Probabilistic Models of Proteins and Nucleic Acids. Cambridge University Press, Cambridge.

[vbad052-B4] Eddy S.R. (2011) Accelerated profile HMM searches. PLoS Comput. Biol., 7, e1002195. 22039361 10.1371/journal.pcbi.1002195 PMC3197634

[vbad052-B5] Edgar R.C. (2004) MUSCLE: multiple sequence alignment with high accuracy and high throughput. Nucleic Acids Res., 32, 1792–1797. 15034147 10.1093/nar/gkh340 PMC390337

[vbad052-B6] Fletcher W. , Yang Z. (2009) INDELible: a flexible simulator of biological sequence evolution. Mol. Biol. Evol., 26, 1879–1888. 19423664 10.1093/molbev/msp098 PMC2712615

[vbad052-B7] Garriga E. et al (2019) Large multiple sequence alignments with a root-to-leaf regressive method. Nat. Biotechnol., 37, 1466–1470. 31792410 10.1038/s41587-019-0333-6 PMC6894943

[vbad052-B8] Katoh K. , Frith M.C. (2012) Adding unaligned sequences into an existing alignment using MAFFT and LAST. Bioinformatics, 28, 3144–3146. 23023983 10.1093/bioinformatics/bts578 PMC3516148

[vbad052-B9] Katoh K. et al (2002) MAFFT: a novel method for rapid multiple sequence alignment based on fast Fourier transform. Nucleic Acids Res., 30, 3059–3066. 12136088 10.1093/nar/gkf436 PMC135756

[vbad052-B10] Matsen F.A. et al (2010) pplacer: linear time maximum-likelihood and Bayesian phylogenetic placement of sequences onto a fixed reference tree. BMC Bioinformatics, 11, 1–16. 21034504 10.1186/1471-2105-11-538 PMC3098090

[vbad052-B11] Mirarab S. , Warnow T. (2011) FastSP: linear time calculation of alignment accuracy. Bioinformatics, 27, 3250–3258. 21984754 10.1093/bioinformatics/btr553

[vbad052-B12] Mirarab S. , Warnow T. (2015) ASTRAL-II: coalescent-based species tree estimation with many hundreds of taxa and thousands of genes. Bioinformatics, 31, i44–i52. 26072508 10.1093/bioinformatics/btv234 PMC4765870

[vbad052-B13] Mirarab S. et al (2015) PASTA: Ultra-Large Multiple Sequence Alignment for Nucleotide and Amino-Acid Sequences. J. Comput. Biol., 22, 377–386. 25549288 10.1089/cmb.2014.0156 PMC4424971

[vbad052-B14] Morrison D.A. (2006) Multiple sequence alignment for phylogenetic purposes. Aust. Syst. Bot., 19, 479–539.

[vbad052-B15] Nguyen N.-P.D. et al (2015) Ultra-large alignments using phylogeny-aware profiles. Genome Biol., 16, 1–15. 26076734 10.1186/s13059-015-0688-z PMC4492008

[vbad052-B16] Nute M. , Warnow T. (2016) Scaling statistical multiple sequence alignment to large datasets. BMC Genomics, 17, 135–144. 28185555 10.1186/s12864-016-3101-8 PMC5123300

[vbad052-B17] Park M. , Warnow T. (2023) [Dataset] INDELible simulated datesets with sequence length heterogeneity. Illinois Data Bank. 10.13012/B2IDB-0900513_V1.

[vbad052-B18] Price M.N. et al (2010) FastTree 2 – approximately maximum-likelihood trees for large alignments. PLoS ONE, 5, e9490. 20224823 10.1371/journal.pone.0009490 PMC2835736

[vbad052-B19] Shen C. et al (2022) WITCH: improved multiple sequence alignment through weighted consensus HMM alignment. J. Comput. Biol., 29, 782–801. 35575747 10.1089/cmb.2021.0585

[vbad052-B20] Shendure J. , Ji H. (2008) Next-generation DNA sequencing. Nat. Biotechnol., 26, 1135–1145. 18846087 10.1038/nbt1486

[vbad052-B21] Sievers F. et al (2011) Fast, scalable generation of high-quality protein multiple sequence alignments using Clustal Omega. Mol. Syst. Biol., 7, 539. 21988835 10.1038/msb.2011.75 PMC3261699

[vbad052-B22] Smirnov V. , Warnow T. (2021a) MAGUS: multiple sequence alignment using graph clUStering. Bioinformatics, 37, 1666–1672. 33252662 10.1093/bioinformatics/btaa992 PMC8289385

[vbad052-B23] Smirnov V. , Warnow T. (2021b) Phylogeny estimation given sequence length heterogeneity. Syst. Biol., 70, 268–282. 32692823 10.1093/sysbio/syaa058 PMC7875441

[vbad052-B24] Stamatakis A. (2014) RAxML version 8: a tool for phylogenetic analysis and post-analysis of large phylogenies. Bioinformatics, 30, 1312–1313. 24451623 10.1093/bioinformatics/btu033 PMC3998144

[vbad052-B25] Stoye J. et al (1998) Rose: generating sequence families. Bioinformatics, 14, 157–163. 9545448 10.1093/bioinformatics/14.2.157

[vbad052-B26] Suchard M.A. , Redelings B.D. (2006) Bali-Phy: simultaneous Bayesian inference of alignment and phylogeny. Bioinformatics, 22, 2047–2048. 16679334 10.1093/bioinformatics/btl175

