# Supplementary material for: HMMerge: an ensemble method for multiple sequence alignment
Source: Bioinform Adv. 2023 Apr 17;3(1):vbad052. doi: 10.1093/bioadv/vbad052 (PMC10148686; doi:10.1093/bioadv/vbad052)
Supplement: vbad052_Supplementary_Data [file vbad052_supplementary_data.pdf]

Supplementary Materials - HMMerge: an Ensemble Method for Multiple Sequence Alignment

Minhyuk Park and Tandy Warnow

Contents

S1 Commands and Versions of Software Used 3
- S1.1 UPP . . . 3
- S1.2 MAGUS . . . 3
- S1.3 PASTA . . . 3
- S1.4 MAFFT . . . 3
- S1.5 MAFFT addfrags . . . 3
- S1.6 Clustal Omega . . . 4
- S1.7 MUSCLE . . . 4
- S1.8 FastSP . . . 4
- S1.9 HMMerge . . . 4
- S1.10WITCH . . . 4
- S1.11Additional details . . . 5

S2 Additional Tables 6

S3 Additional Figures 8

S4 Impact of larger eHMM 22

S5 Scalability of HMMerge 24

List of Tables

- S1 Simulated DNA/RNA dataset overview . . . 6
- S2 Biological DNA/RNA dataset overview . . . 7
- S3 Impact of Adding to the eHMM in HMMerge . . . 7

List of Figures

- S1 Total alignment error for seven benchmark methods on ROSE simulated datasets with introduced fragmentation . . . 8
- S2 Query-only sequence alignment error of UPP, WITCH, and HMMerge . . . 9
- S3 Total alignment error for HMMerge, WITCH, UPP, and MAGUS on ROSE simulated datasets with introduced fragmentation . . . 10

S4 Total alignment error of ten benchmark methods on INDELible simulated datasets with introduced fragmentation . . . 11
S5 Query-only alignment error for MAFFT-addfrags, UPP, WITCH, and HMMerge on RNASim1000 simulated datasets with introduced fragmentation . . . 12
S6 Total alignment error of ten benchmark methods on RNASim1000 simulated datasets with introduced fragmentation . . . 13
S7 Query-only alignment error of UPP, WITCH, HMMerge, and MAFFT-addfrags on CRW biological datasets . . . 14
S8 Total alignment error of ten benchmark methods on CRW biological datasets . . . 15
S9 Impact of backbone alignment method on the query-only alignment error of MAFFT-addfrags 16
S10 ROSE Simulated Datasets Sequence Length Histograms . . . 17
S11 INDELible Simulated Datasets Sequence Length Histograms . . . 18
S12 RNASim Simulated Datasets Sequence Length Histograms . . . 19
S13 CRW 23S Biological Datasets Sequence Length Histograms . . . 20
S14 CRW 5S Biological Datasets Sequence Length Histograms . . . 21
S15 Illustration of jumping between base HMMs . . . 23

S1 Commands and Versions of Software Used

S1.1 UPP

Version: 4.5.2
Availability: https://github.com/smirarab/sepp

```
python run_upp.py -c <Config file>
```

```
[commandline]
tree=<FastTree backbone tree>
alignment=<MAGUS backbone alignment>
sequence_file=<Unaligned query sequence file>
backboneSize=<backbone size>
alignmentSize=2
molecule=dna
cpu=16
outdir=<Output directory>
tempdir=<Temporary output directory>
```

S1.2 MAGUS

Version: 0.1.0b2
Availability: https://github.com/vlasmirnov/MAGUS

```
python <git root>/magus.py -d <Output directory> -i \
<Unaligned sequences> -o <Output filename>
```

S1.3 PASTA

Version: 1.9.0
Availability: https://github.com/smirarab/pasta

```
python run_pasta.py -i <Unaligned sequences> --num-cpus 16 \
-o <Output directory> --temporaries <Temporary output directory>
```

S1.4 MAFFT

Version: 7.487
Availability: https://mafft.cbrc.jp/alignment/software/linux.html

```
linsi --thread 16 <Unaligned sequences> 1> <Output filename>
```

S1.5 MAFFT addfrags

Version: 7.487
Availability: https://mafft.cbrc.jp/alignment/software/linux.html

```
mafft --addfragments --thread 16 <Unaligned sequences> 1> <Output filename>
```

S1.6 Clustal Omega

Version: 1.2.4
Availability: http://www.clustal.org/omega/#Download

```
clustalo --threads=16 --in <Unaligned sequences> --out \
<Output filenanme>
```

T-COFFEE Version: 13.45.0.4846264
Availability: https://www.tcoffee.org/Packages/Stable/Latest/

```
t_coffee -thread=16 -reg -seq <Unaligned sequences> -outfile \
<Output filename>
```

S1.7 MUSCLE

Version: 3.8.31
Availability: https://drive5.com/muscle/downloads_v3.htm

```
muscle -in <Unaligned sequences> -out <Output filename>
```

S1.8 FastSP

Version: 1.7.1
Availability: https://github.com/smirarab/FastSP
Note: When evaluating MAFFT estimated alignments, the ml and mlr flags should be omitted.

```
java -jar FastSP.jar -r <Reference alignment> -e \
<Estimated alignment> -ml -mlr
```

S1.9 HMMerge

Commit ID: 741d1d869fc1d1dbeb645707478f755684b9c739
Availability: https://github.com/MinhyukPark/HMMerge

```
python <git root>/main.py --input-dir <Directory with \
partitioned backbone alignments> --backbone-alignment \
<Backbone alignment> --query-sequence-file \
<Query sequences> --output-prefix <Output directory> \
--num-processes 16 --model DNA
```

S1.10 WITCH

Commit ID: a5fff8b8e9491869a151318061543a2af22db7df
Availability: https://github.com/c5shen/WITCH

```
python <git root>witch.py -t 16 -b <Backbone alignment> \
-e <Backbone tree> -q <Query sequences> -d \
<Output directory> --molecule dna -o <Output filename>
```

S1.11 Additional details

WITCH, UPP, and HMMerge all share the same algorithm for decomposing an input alignment into subsets. This centroid edge decomposition algorithm is described in the main paper. In summary, the input backbone tree is recursively subdivided at a centroid edge until the subset created falls below a certain size threshold. WITCH and UPP keep the subsets created at all levels of decomposition, creating a hierarchical set of HMMs, while HMMerge in this study only kept the leaf level subsets, creating a disjoint set of HMMs.

WITCH and UPP, in fact, share the same exact code for decomposing the backbone alignment. In this study, WITCH decomposed the input alignments down to subsets of size 10 while UPP decomposed the input alignments down to subsets of size 2. The decomposed subsets passed to HMMerge were obtained through a modification of the decomposition code that was used in PASTA [3].

S2 Additional Tables

Table S1: **Simulated DNA/RNA dataset overview** Here, we show the basic empirical statistics about the datasets used in this study. All datasets have 1000 sequences. Length is the length of the true alignment averaged over the replicates. The first 15 rows are ROSE model conditions, which have 20 replicates each; the remaining rows have 10 replicates each. Results shown are averaged across the replicates. The p-distances refer to the normalized Hamming distance, computed prior to fragmentation.

Name	Length	% gap	# Full	# Query	avg. p-dist.	max. p-dist.	avg gap len.	med gap len.
1000S1	2141.2	0.53	500	500	0.694	0.768	4.0	3.4
1000S2	1546.0	0.35	500	500	0.693	0.768	2.9	2.4
1000S3	1595.2	0.37	500	500	0.686	0.763	2.9	2.4
1000S4	1328.1	0.25	500	500	0.501	0.608	2.5	2.0
1000S5	1165.2	0.14	500	500	0.498	0.611	2.3	2.0
1000M1	3965.0	0.74	500	500	0.695	0.769	10.1	8.0
1000M2	3972.3	0.74	500	500	0.684	0.762	10.3	7.9
1000M3	2722.6	0.63	500	500	0.660	0.741	7.6	5.6
1000M4	2570.6	0.61	500	500	0.495	0.606	7.6	5.8
1000M5	1810.0	0.44	500	500	0.499	0.602	6.2	4.4
1000L1	3817.5	0.73	500	500	0.695	0.769	13.6	10.7
1000L2	2406.9	0.58	500	500	0.696	0.769	11.6	9.3
1000L3	7042.8	0.85	500	500	0.687	0.763	20.0	16.1
1000L4	2446.2	0.59	500	500	0.500	0.608	11.4	9.2
1000L5	1764.8	0.43	500	500	0.496	0.606	10.4	8.0
RNASim1000	21,946.0	0.96	500	500	0.409	1.000	21.6	9.0
IND. 0.001	442,255.6	0.74	500	500	0.613	0.756	6.5	4.6
IND. 0.005	923,289.0	0.93	500	500	0.613	0.761	15.6	12.6

Table S2: **Biological DNA/RNA dataset overview** Here, we show the basic empirical statistics about the biological datasets used in this study. The p-distances refer to the normalized Hamming distance. L(bp) refers to the length of the reference alignments, which are curated and provided by [1]. Percent gapped refers to the percent of the reference alignment that has dashes rather than letters. For these biological datasets, the backbone sequences were chosen by partitioning the datasets at length 1250 for 23S.C and 23S.A and at length 100 for 5S.3, 5S.E, and 5S.T.

Name	# Sequences	L(bp)	% gap	# Full	# Query	avg. p-dist.	max. p-dist.	avg gap len.	median gap len.
23S.A	214	3991	0.54	133	81	0.293	0.667	8.0	1.0
23S.C	374	5916	0.65	274	100	0.143	0.750	10.3	1.0
5S.3	5507	414	0.74	4439	1068	0.417	1.000	5.1	1.0
5S.E	2774	793	0.88	1886	888	0.305	1.000	14.3	1.0
5S.T	5751	436	0.76	4677	1074	0.425	1.000	64.0	2.0

	Disjoint 50	Disjoint 50 + BB	UPP 50	UPP 10
5S.3 Average	0.106	0.106	0.102	0.105
5S.3 SPFN	0.129	0.129	0.125	0.128
5S.3 SPFP	0.084	0.084	0.078	0.081
5S.E Average	0.091	0.091	0.089	0.083
5S.E SPFN	0.103	0.103	0.105	0.092
5S.E SPFP	0.079	0.079	0.072	0.073
5S.T Average	0.106	0.106	0.096	0.097
5S.T SPFN	0.122	0.122	0.111	0.111
5S.T SPFP	0.090	0.090	0.081	0.083
Indelible-0.001-HF Average	0.095	0.095	NR	NR
Indelible-0.001-HF SPFN	0.107	0.107	NR	NR
Indelible-0.001-HF SPFP	0.084	0.084	NR	NR
ROSE-1000S1-HF Average	0.148	0.148	NR	NR
ROSE-1000S1-HF SPFN	0.156	0.156	NR	NR
ROSE-1000S1-HF SPFP	0.141	0.141	NR	NR

Table S3: **Impact of Adding to the eHMM on the Query-Only Alignment Error** Disjoint 50 is the default ensemble of HMMerge where only the HMMs for the minimal sequence sets are included in the eHMM for HMMerge (the sequence sets are disjoint). In Disjoint 50 + BB, we add the 1 HMM built on the entire backbone alignment to the ensemble. UPP N use a hierarchical ensemble of HMMs, and the decomposition stops when all subsets are of size at most N. “NR” means that the analysis was not run.

S3 Additional Figures

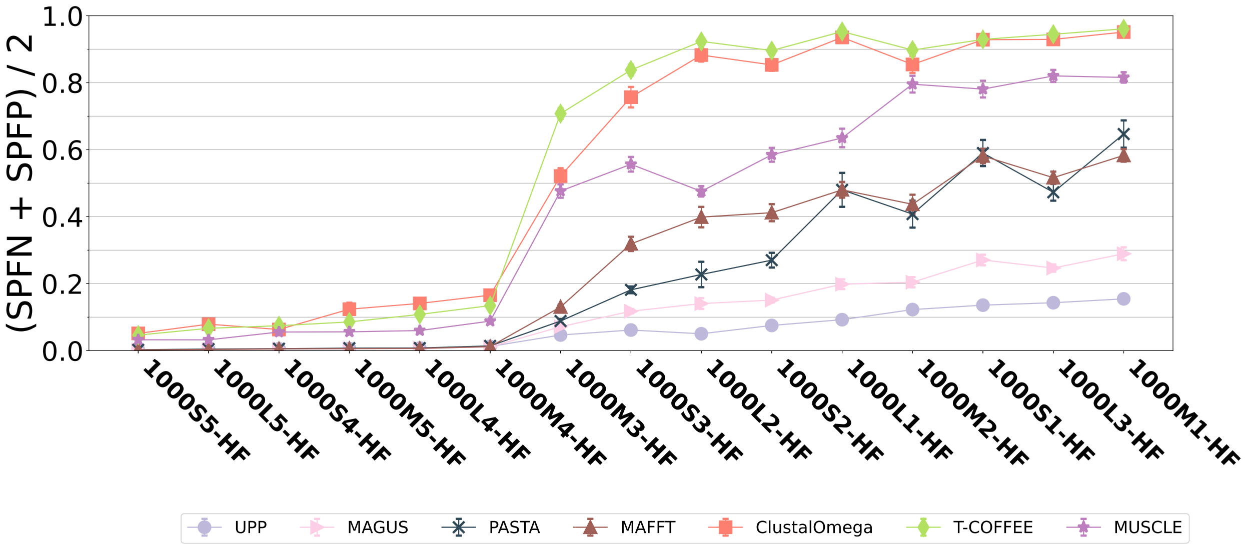

Figure S1: **Total alignment error for seven benchmark methods on ROSE simulated datasets with introduced fragmentation** Alignment error (average of SPFN and SPFP) of all of the sequences of the final alignment produced by each method. These are highly fragmented ("HF" for short) datasets created from ROSE simulated datasets introduced in the SATé study [2]. All datasets have 1000 sequences, with substitution rates that roughly increase from left-to-right. The error bars indicate standard error over 20 replicates. MAFFT here is MAFFT-linsi.

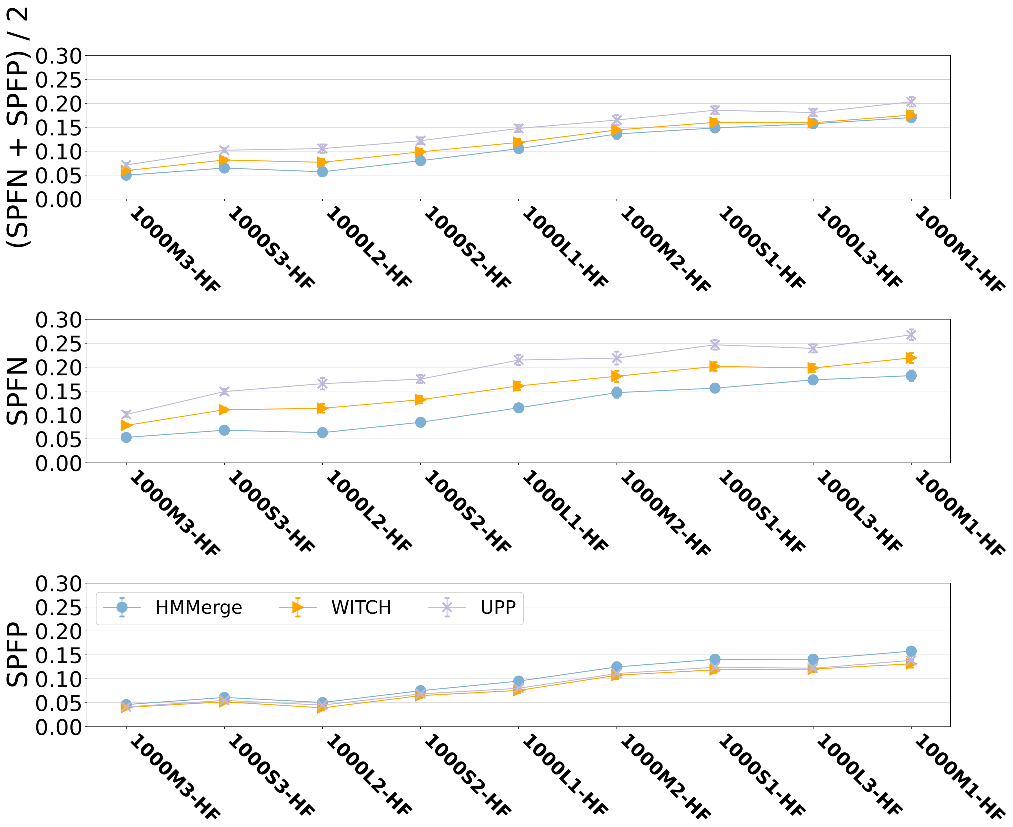

Figure S2: **Query-only sequence alignment error of UPP, WITCH, and HMMerge on ROSE simulated datasets with introduced fragmentation**. We show the average, SPFN, and SPFP errors on the query sequences of the alignment produced by UPP, WITCH, and HMMerge on the ROSE simulated datasets with introduced fragmentation. These datasets have 20 replicates; error bars are standard error.

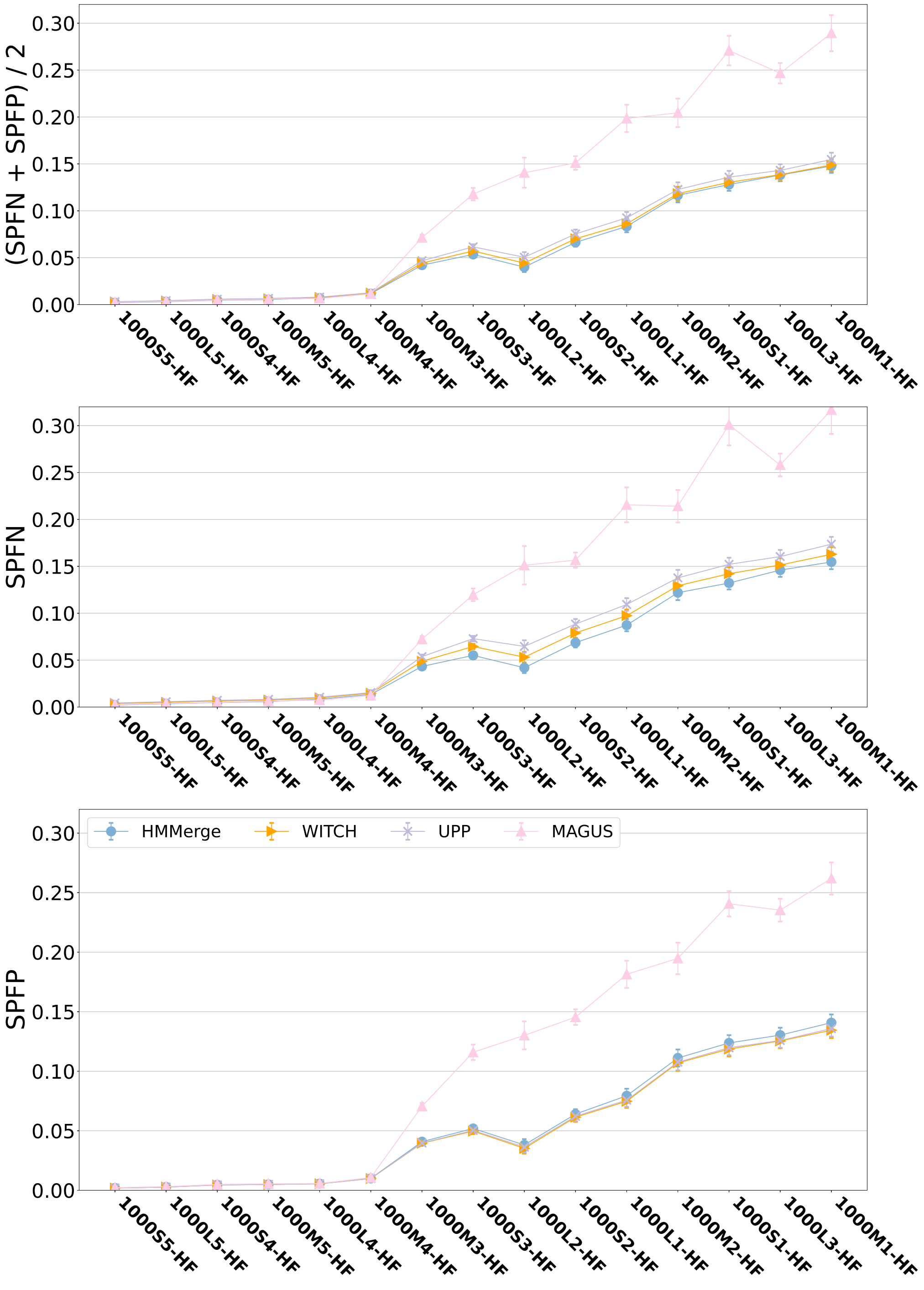

Figure S3: **Total alignment error for HMMerge, WITCH, UPP, and MAGUS on ROSE simulated datasets with introduced fragmentation** We show the average, SPFN, and SPFP errors on all of the sequences of the final alignments produced by HMMerge, WITCH, UPP, and MAGUS on the ROSE simulated datasets with introduced fragmentation. The error bars indicate standard error over 20 replicates.

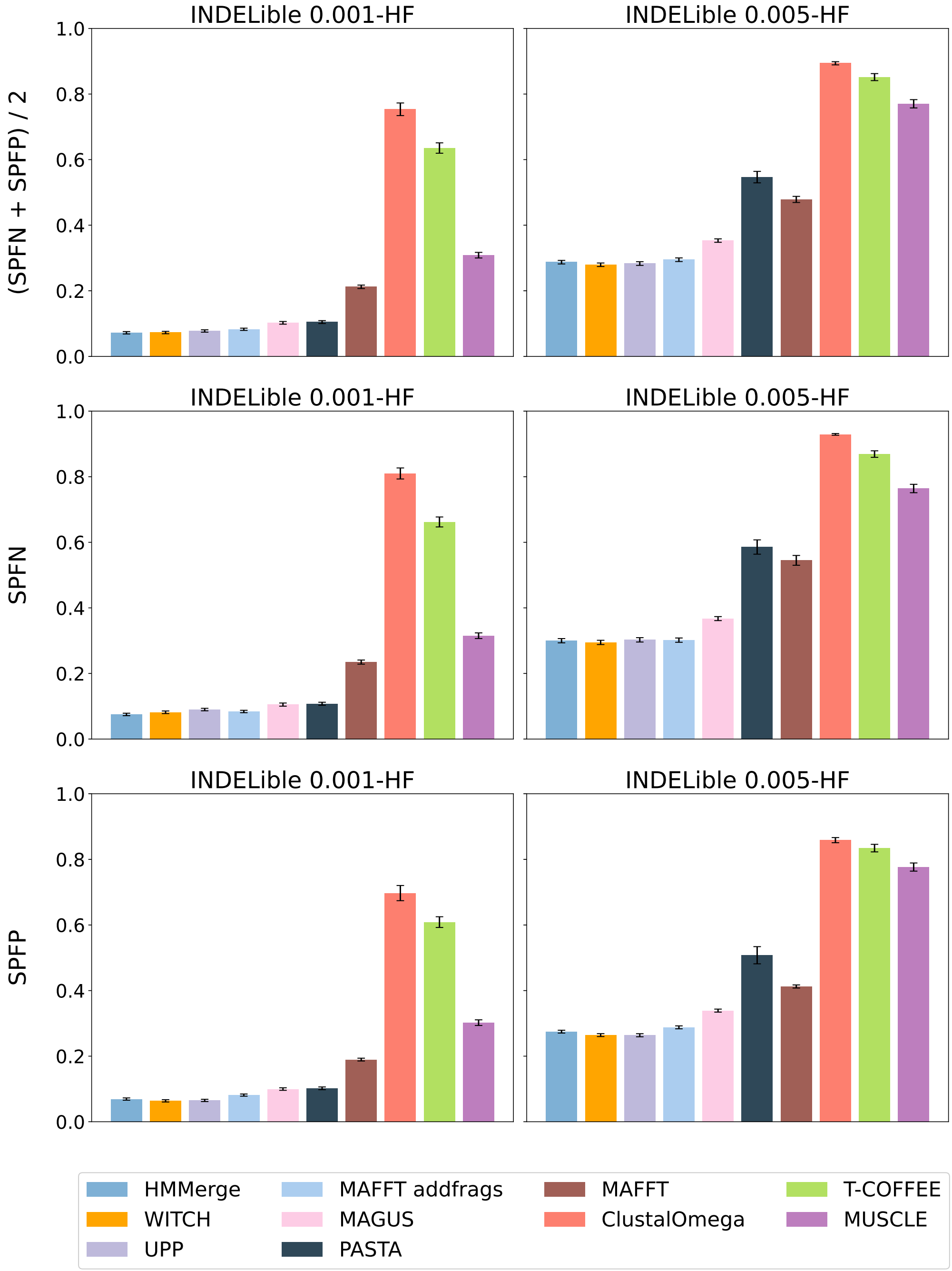

Figure S4: **Total alignment error of ten benchmark methods on INDELible simulated datasets with introduced fragmentation** We show the average, SPFN, and SPFP errors on all of the sequences of the alignment produced by ten benchmark methods on the INDELible simulated datasets 0.001-HF and 0.005-HF. The INDELible datasets have 10 replicates, and error bars show standard error. MAFFT here is MAFFT-linsi.

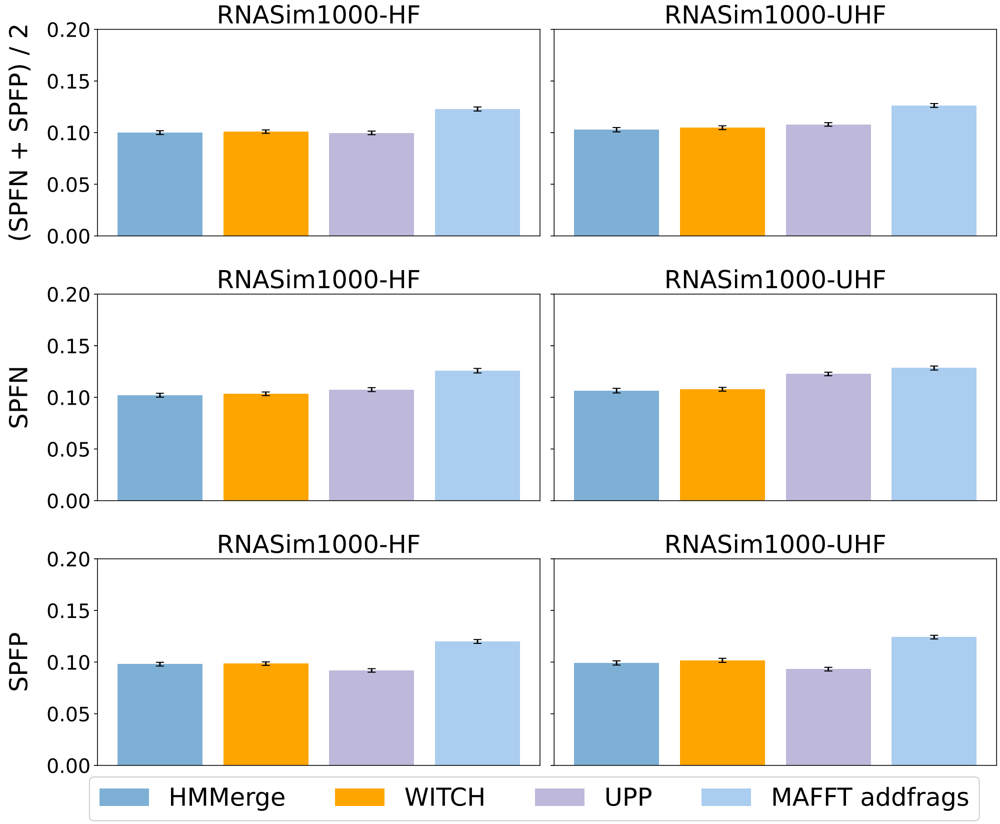

Figure S5: **Query-only alignment error for MAFFT-addfrags, UPP, WITCH, and HMMerge on RNASim1000 simulated datasets with introduced fragmentation** The RNASim datasets have 10 replicates, and error bars show standard error.

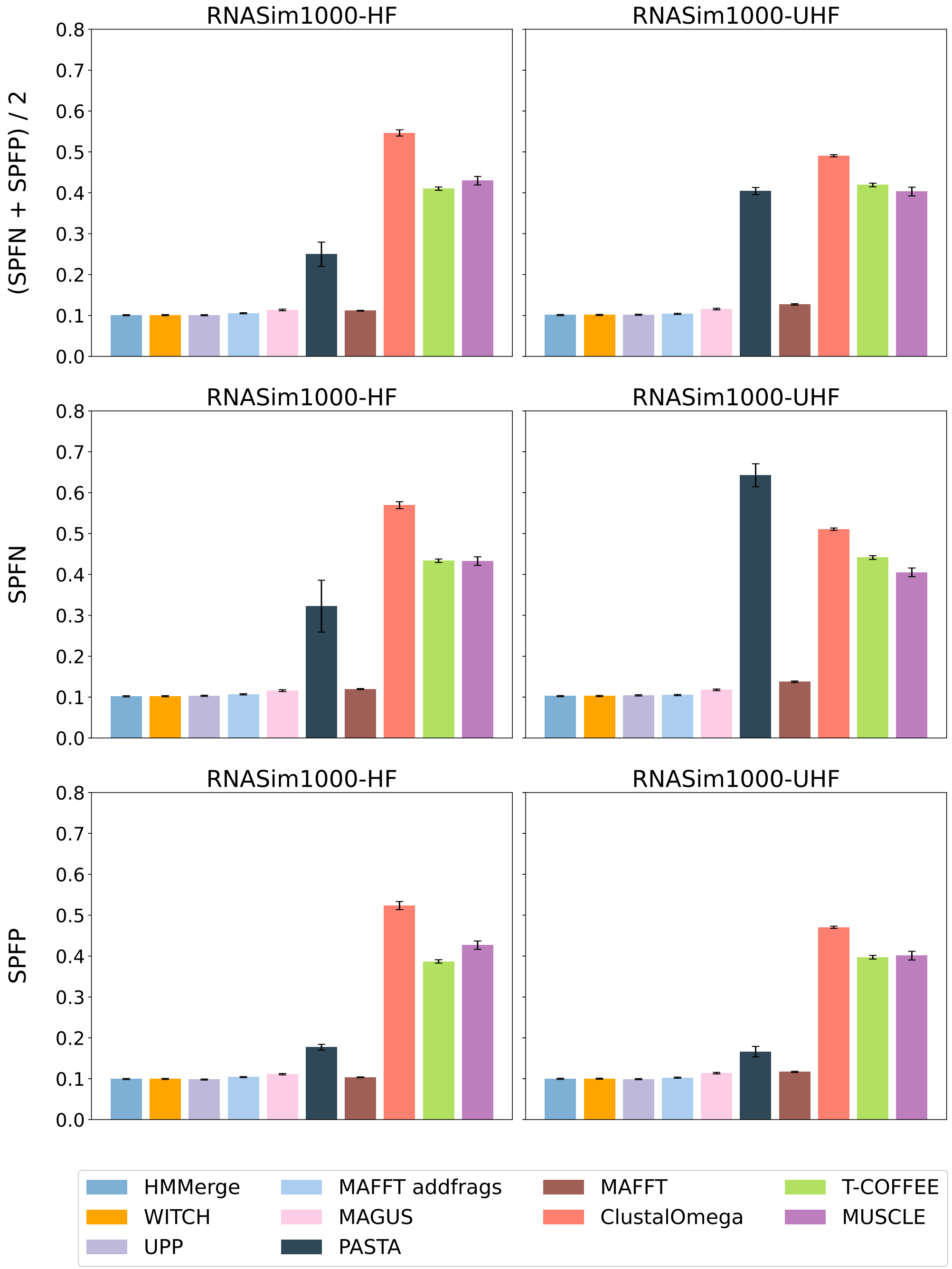

Figure S6: **Total alignment error of ten benchmark methods on RNASim1000 simulated datasets with introduced fragmentation** We show the average, SPFN, and SPFP errors on all of the sequences of the final alignments produced by different methods. The error bars indicate standard error over 10 replicates. MAFFT here is MAFFT-linsi.

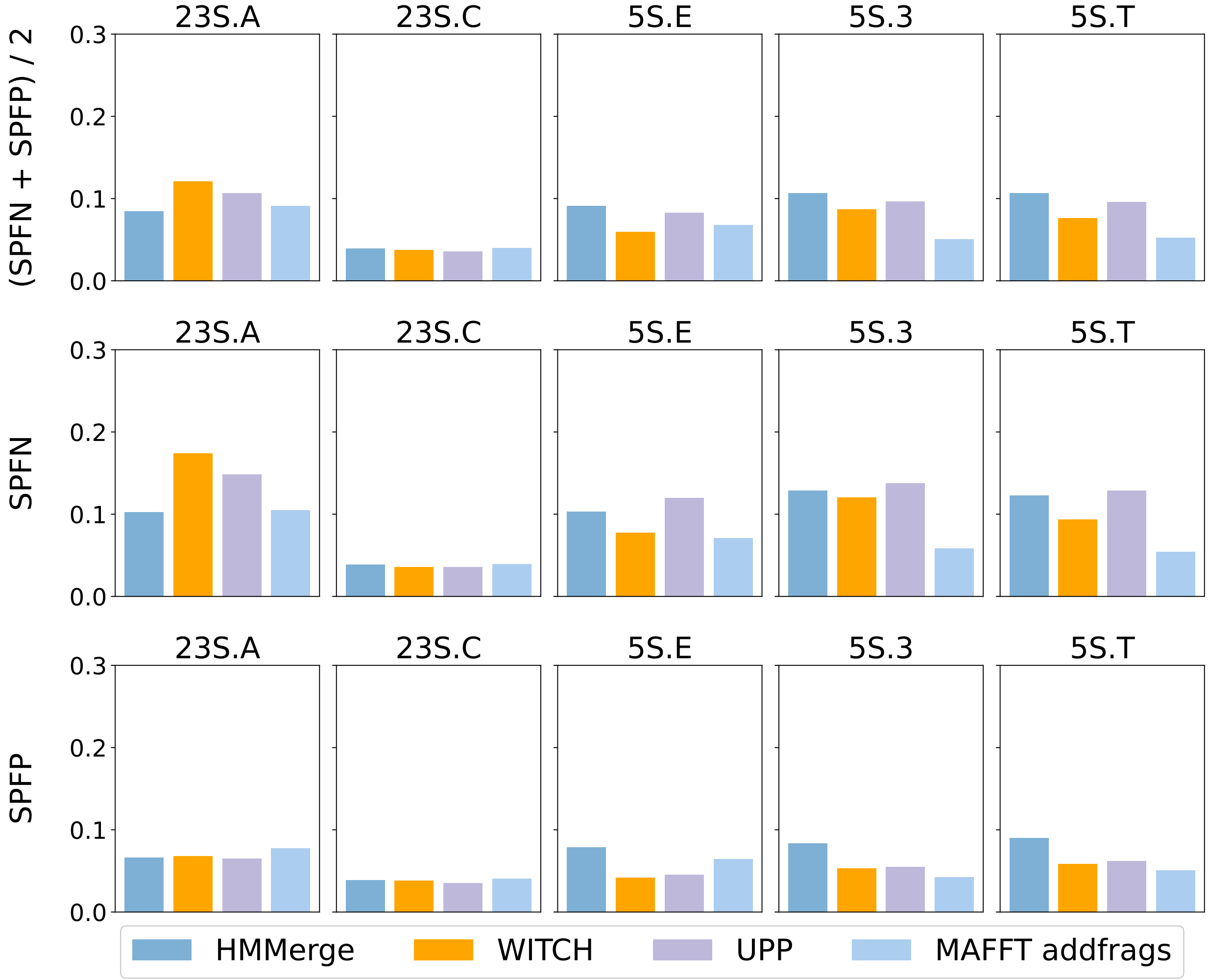

Figure S7: **Query-only alignment error of UPP, WITCH, HMMerge, and MAFFT-addfrags on CRW biological datasets** Each dataset is a single replicate, so there are no error bars.

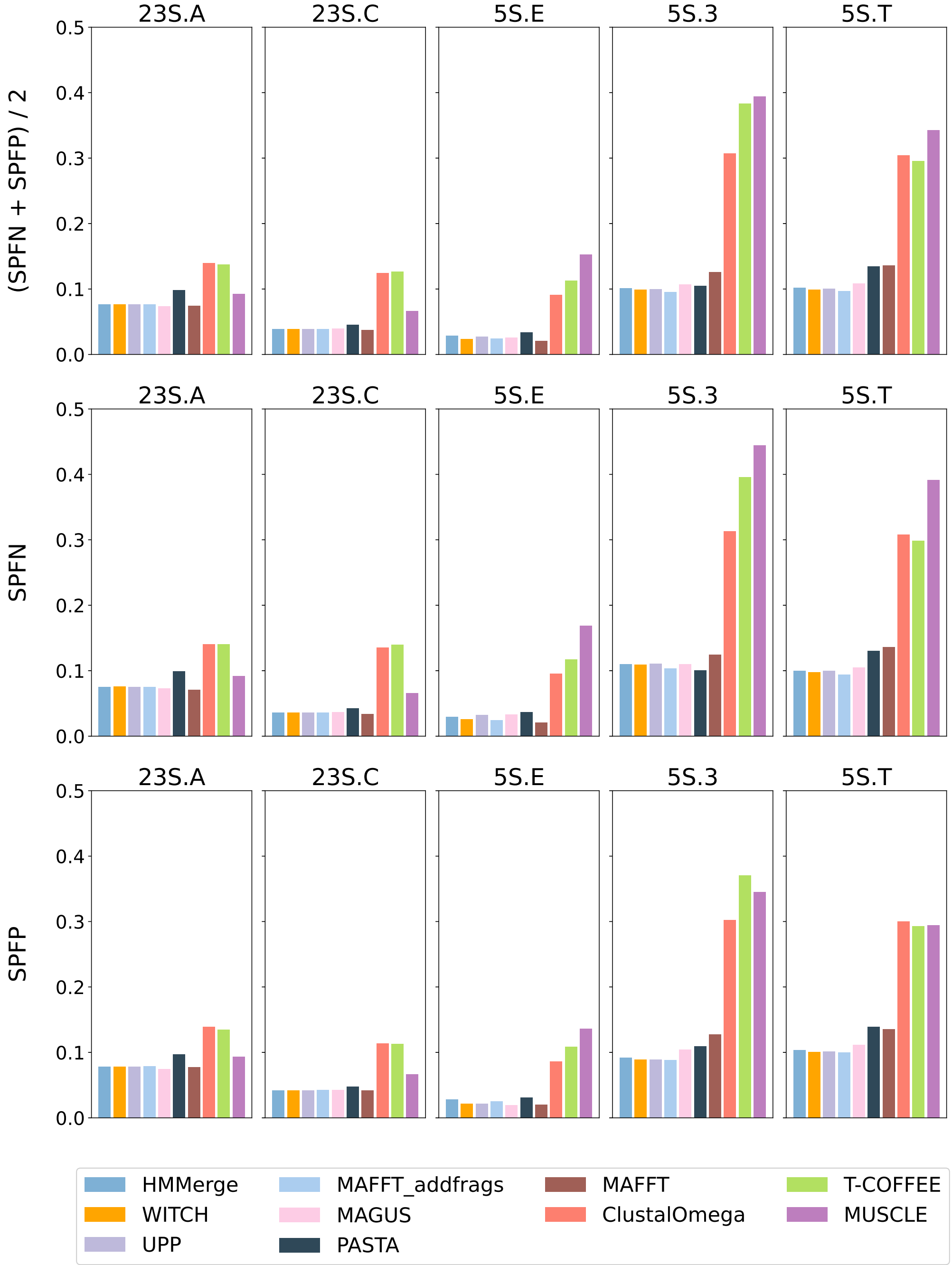

Figure S8: **Total alignment error of ten benchmark methods on CRW biological datasets** Each dataset is a single replicate, so there are no error bars.

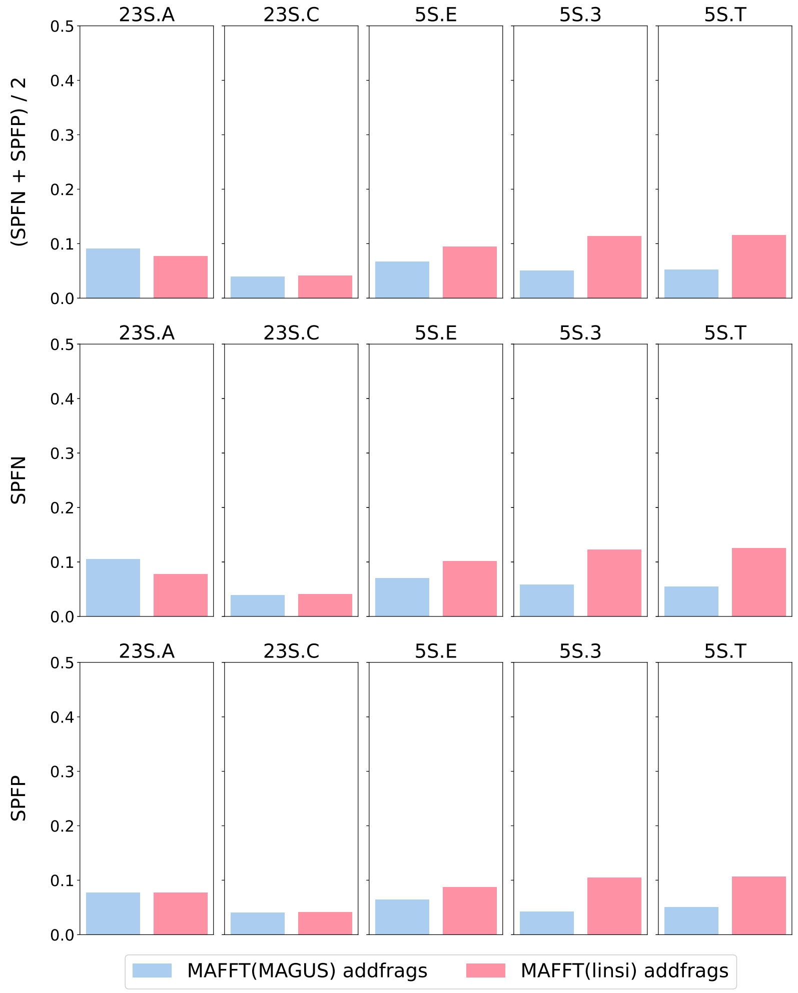

Figure S9: **Impact of backbone alignment method on the query-only alignment error of MAFFT-addfrags.** We show the alignment errors (average, SPFN, and SPFP) of MAFFT-addfrags algorithm with two different backbone alignment methods: MAGUS and MAFFT-linsi. Although the impact depends on the dataset, overall using the MAGUS backbone is generally favorable. Each dataset is a single replicate, so there are no error bars.

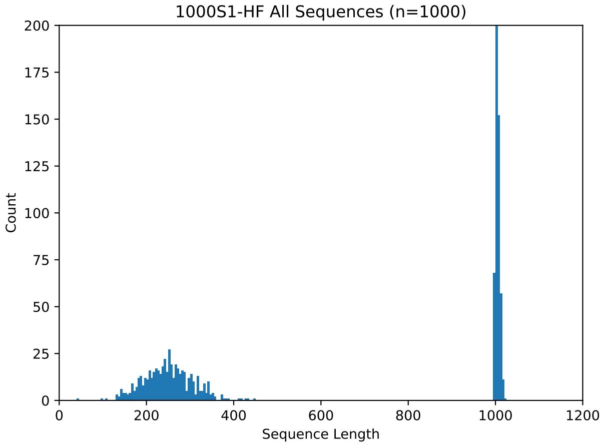

(a) ROSE 1000S1-HF sequence length histogram

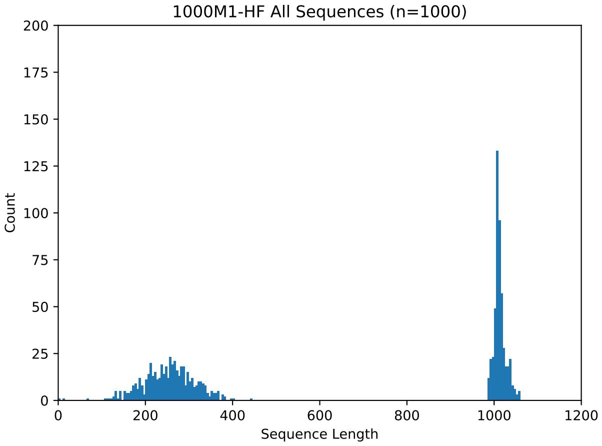

(b) ROSE 1000M1-HF sequence length histogram

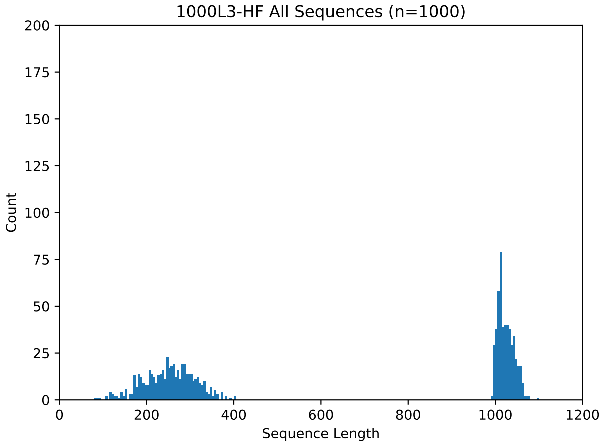

(c) ROSE 1000L3-HF sequence length histogram

Figure S10: ROSE Simulated Datasets Sequence Length Histograms

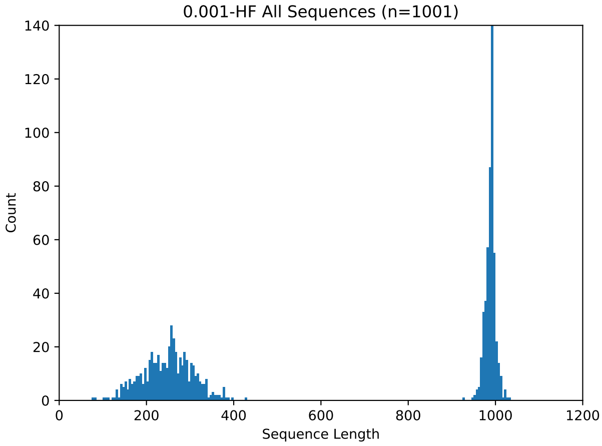

(a) INDELible 0.001-HF sequence length histogram

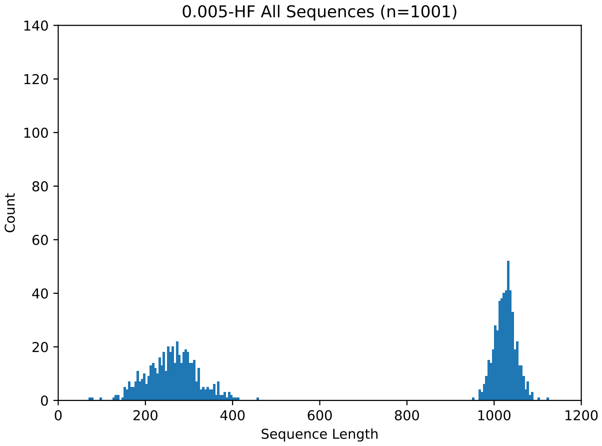

(b) INDELible 0.005-HF sequence length histogram

Figure S11: INDELible Simulated Datasets Sequence Length Histograms

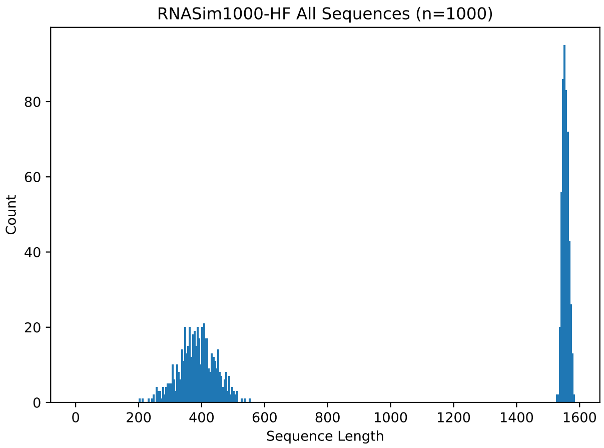

(a) RNASim1000-HF sequence length histogram

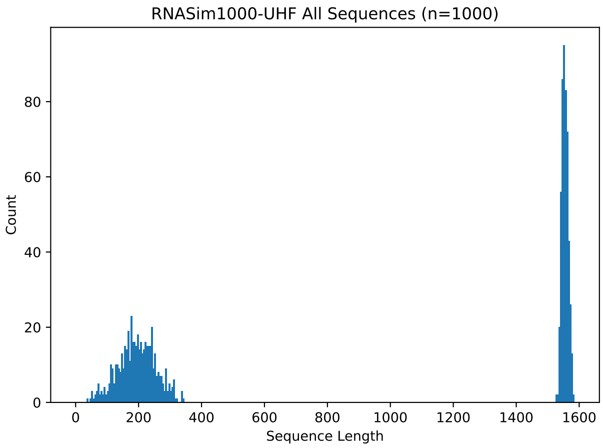

(b) RNASim1000-UHF sequence length histogram

Figure S12: RNASim Simulated Datasets Sequence Length Histograms

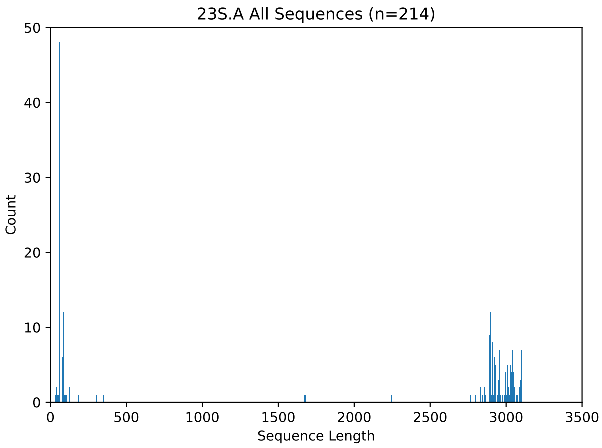

(a) 23S.A sequence length histogram

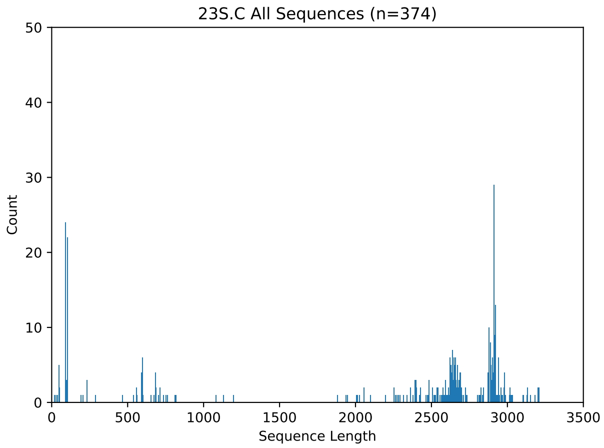

(b) 23S.C sequence length histogram

Figure S13: CRW 23S Biological Datasets Sequence Length Histograms

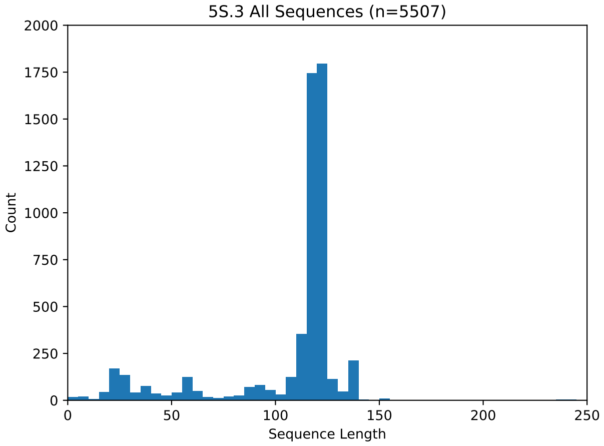

(a) 5S.3 sequence length histogram

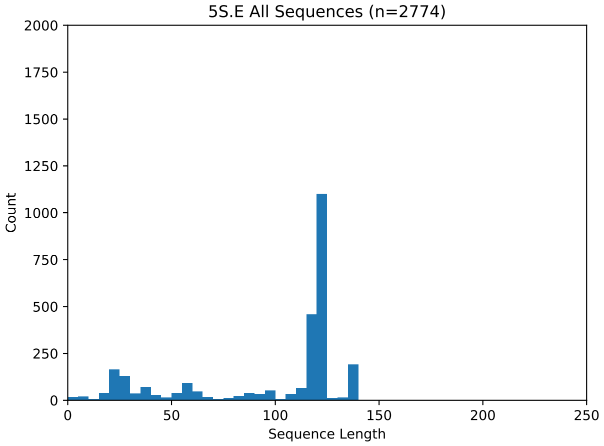

(b) 5S.E sequence length histogram

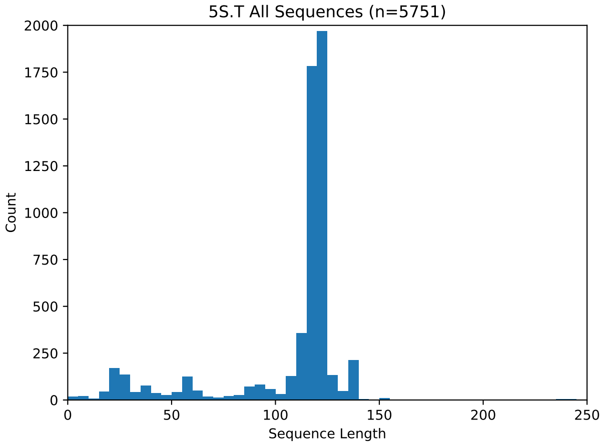

(c) 5S.T sequence length histogram

Figure S14: CRW 5S Biological Datasets Sequence Length Histograms

S4 Impact of larger eHMM

In this section we explore the impact of using larger eHMMs. The default usage has HMMs on the minimal alignment subsets only from the UPP(50) ensemble, but here we explore the impact of including also the HMM on the full backbone alignment, or going further and including all the HMMs in the UPP(50) ensemble.

We show in Figure S15 that including the HMM for the full backbone alignment in the eHMM enables jumping between HMMs, which results in improved accuracy.

In contrast, in our studies on biological and simulated datasets where we added the HMM for the full backbone (see Table S3), we did not see any difference in the alignment error; this suggests that this extreme case given in our figure does not occur in these datasets. On the other hand, using the UPP(50) eHMM, which includes every HMM ever created during the hierarchical decomposition rather than just the HMMs for the minimal sequence sets, did sometimes improve accuracy (see Table S3). This observation is consistent with studies in [4], which explored UPP with changes to the eHMM (including using only the HMMs for the minimal sequence sets, as used here). Thus, there is likely room for improvement in accuracy for HMMerge through using larger eHMMs.

```
# input subalignment 1
AAAA----GGGGG
AAAA----GGGGG
AAAA----GGGGG

# input subalignment 2
----TTTTGGGGG
----TTTTGGGGG
----TTTTGGGGG

# backbone alignment
AAAA----GGGGG
AAAA----GGGGG
AAAA----GGGGG
----TTTTGGGGG
----TTTTGGGGG
----TTTTGGGGG

# input query sequence
AAAATTTTGGGGG

# output alignment
AAAA----GGGG
AAAA----GGGG
AAAA----GGGG
----TTTTGGGG
----TTTTGGGG
----TTTTGGGG
AAAATTTTGGGG
```

(a) **Using HMM for Backbone Alignment in Input allows jumping**

```
# input subalignment 1
AAAA----GGGGG
AAAA----GGGGG
AAAA----GGGGG

# input subalignment 2
----TTTTGGGGG
----TTTTGGGGG
----TTTTGGGGG

# input query sequence
AAAATTTTGGGGG

# output alignment
----AAAA----GGGG
----AAAA----GGGG
----AAAA----GGGG
--------TTTTGGGG
--------TTTTGGGG
--------TTTTGGGG
aaaa----TTTTGGGG
```

(b) **Not including HMM for Backbone Alignment in Input does not enable jumping**

Figure S15: **Illustration of Jumping Between Base HMMs.** In subfigure (a), we show an example of HMMerge selecting three HMMs for its eHMM: two on sub-alignments and also the backbone alignment as input. When merging the three HMMs built on these three input alignments, several edges are created. For example, from input subalignment 1, edges from column 3 to column 8 are created (zero indexed). Most notably, from the backbone alignment, edges from column 3 to column 4 are created (also zero indexed). This allows the input query sequence to be aligned to the HMM built on input subalignment 1 for the string of As and then transition to input subalignment 2 using the edges created by the backbone alignment. This "jumping" is impossible in subfigure (b) where no input subalignments provide edges to go from the region of As We to the region of Ts. This is why the output alignment of subfigure (b) can only align one of the regions.

S5 Scalability of HMMerge

Although HMMerge was able to complete on the datasets we studied, which ranged up to 5751 sequences for the 5S.T dataset, we found that HMMerge required more memory and a longer runtime than WITCH, and WITCH itself required a longer runtime than UPP. Furthermore, some HMMerge analyses we attempted, such as aligning datasets using the entire UPP(50) ensemble, required more memory than was available to us in the University of Illinois Campus Cluster queue, which typically have at most 64GB, and some analyses exceeded the four-hour time limit. For these compute-intensive analyses, we had to use a high memory machine with up to 1TB of memory and which allowed longer analyses. For example, HMMerge was given up to 512 GB of memory on some HMMerge runs on the INDELible datasets, which have long sequences. In general, HMMerge did not require more than 512GB of memory except for some runs of experiments that used the entire UPP(50) ensemble where HMMerge was given up to 1TB.

References

[1] Jamie J Cannone, Sankar Subramanian, Murray N Schnare, James R Collett, Lisa M D'Souza, Yushi Du, Brian Feng, Nan Lin, Lakshmi V Madabusi, Kirsten M Müller, et al. The Comparative RNA Web (CRW) Site: an online database of comparative sequence and structure information for ribosomal, intron, and other RNAs. *BMC Bioinformatics*, 3(1):1–31, 2002.

[2] Kevin Liu, Sindhu Raghavan, Serita Nelesen, C Randal Linder, and Tandy Warnow. Rapid and accurate large-scale coestimation of sequence alignments and phylogenetic trees. *Science*, 324(5934):1561–1564, 2009.

[3] Siavash Mirarab, Nam Nguyen, Sheng Guo, Li-San Wang, Junhyong Kim, and Tandy Warnow. PASTA: Ultra-Large Multiple Sequence Alignment for Nucleotide and Amino-Acid Sequences. *Journal of Computational Biology*, 22(5):377–386, 2015.

[4] Nam-phuong D Nguyen, Siavash Mirarab, Keerthana Kumar, and Tandy Warnow. Ultra-large alignments using phylogeny-aware profiles. *Genome Biology*, 16(1):1–15, 2015.
